# Comparisons between *Arabidopsis thaliana* and *Drosophila melanogaster* in relation to Coding and Noncoding Sequence Length and Gene Expression

**DOI:** 10.1155/2015/269127

**Published:** 2015-05-31

**Authors:** Rachel Caldwell, Yan-Xia Lin, Ren Zhang

**Affiliations:** ^1^School of Biological Sciences, University of Wollongong, Northfields Avenue, Keiraville, Wollongong, NSW 2522, Australia; ^2^National Institute for Applied Statistics Research Australia (NIASRA), School of Mathematics and Applied Statistics, University of Wollongong, Northfields Avenue, Keiraville, Wollongong, NSW 2522, Australia

## Abstract

There is a continuing interest in the analysis of gene architecture and gene expression to determine the relationship that may exist. Advances in high-quality sequencing technologies and large-scale resource datasets have increased the understanding of relationships and cross-referencing of expression data to the large genome data. Although a negative correlation between expression level and gene (especially transcript) length has been generally accepted, there have been some conflicting results arising from the literature concerning the impacts of different regions of genes, and the underlying reason is not well understood. The research aims to apply quantile regression techniques for statistical analysis of coding and noncoding sequence length and gene expression data in the plant, *Arabidopsis thaliana*, and fruit fly, *Drosophila melanogaster*, to determine if a relationship exists and if there is any variation or similarities between these species. The quantile regression analysis found that the coding sequence length and gene expression correlations varied, and similarities emerged for the noncoding sequence length (5′ and 3′ UTRs) between animal and plant species. In conclusion, the information described in this study provides the basis for further exploration into gene regulation with regard to coding and noncoding sequence length.

## 1. Introduction

Advances in high-quality sequencing technologies [1, 2] and large-scale resource datasets [3, 4] have enhanced genomics research. Conducting large-scale sequence comparisons has the advantage of identifying the genetic variation and speciation among organisms [5]. Whole-genome expression experiments have also expanded a new era in bioinformatics analyses [6–9]. Understanding relationships and cross-referencing of expression data to large genome data can now be attained and facilitates a greater insight of organismal complexity and the tightly regulated process of gene expression.

There is a continuing interest in the analysis of gene architecture and gene expression to determine the relationship that may exist [10]. Current investigations on the similarities and differences between plant and animal genome structure have led to a greater understanding in biochemical pathways, genetic mechanisms, sequence structures, and functions [11], and comparative studies are more powerful than studying the sequence of a single genome [5]. Furthermore, control of gene expression has been used as a measurement of variation and is often well conserved between species in the coding sequences. In unicellular organisms such as the yeast* Saccharomyces cerevisiae*, research has found that highly expressed genes tend to have smaller compact protein sizes [12]. Other animal genome studies have found that highly expressed genes have fewer and shorter introns and shorter coding sequences and protein lengths and favour more compactness in highly expressed genes [13, 14]. Previous research, however, is divided in opinion, with highly expressed genes not always being compact in plants. There is evidence that suggests that, in higher plant genomes, highly expressed genes comprise longer introns and primary transcripts [15] in contrast with other research on* Arabidopsis* and rice, finding that highly expressed genes are more compact [16], specifically the lengths of the coding sequence (CDS) [17]. Negative correlation between protein length and gene expression breadth in the plant species* Populus tremula* was also observed [18]. Taken together, these observations suggest that the difference in length in relation to gene expression is not merely due to adaptive evolution but rather has specific biological significance [19].

Significance of noncoding regions is less understood across species compared to the coding regions. A range of genomic studies over the last decade has supported the opinion that there are tightly regulated processes and levels of control in the regulation of gene expression. This has included the untranslated gene regions, notably the 5′ and 3′ untranslated regions (UTRs), which may play the most important role in the regulation of gene expression [20]. A study by Lin and Li revealed a strong negative correlation between the 5′ UTR length and expression correlation with cytosolic ribosomal protein patterns in* S. cerevisiae* and* C. albicans* [21], with highly expressed eukaryotic genes tending to have more compact 5′ UTR regions [22]. A plant study on both* Arabidopsis* and rice also reported negative correlation between expression levels and noncoding sequences (both 5′ and 3′ UTRs) [16].

Statistical approaches, such as quantile regression, are a practical statistical method utilized by many biologists in a range of ecological [23] and bioinformatics [24, 25] studies to investigate the relationships between variables. The advantage of using such a model includes the robustness against outliners and helps obtain a more comprehensive analysis of the relationship between variables by using different measures of central tendency and statistical dispersion. When dealing with sequence length and gene expression data, modelling techniques often have difficulty with this data, due to the data values ranging over several orders of magnitude. It is general practice to log-transform the data, particularly when parametric statistical tests, such as *t*-test, ANOVA, or linear regression, are used. The log function tends to squeeze together the larger values and stretches out the smaller values allowing a better view of the data.

The aim of this study was to apply a quantile regression model to reexamine the correlation of gene region lengths and expression levels of* Arabidopsis* using a different and larger set of gene expression data. The research also extended to another species,* Drosophila melanogaster*, so this study not only expanded objects but also conducted a comparison between plant and animal species.

## 2. Methods

### 2.1. Datasets

Sequence and gene expression data were collected from a selection of publicly accessible databases and websites for each of the plant and animal species.

The* Arabidopsis thaliana* sequence data were downloaded from TAIR website (ftp://ftp.arabidopsis.org/home/tair/Sequences/blast_datasets/TAIR10_blastsets/). The sequence data used were generated from TAIR10 (December 2010) release. Gene expression data were downloaded from the NCBI GEO Datasets database (series GSE31488) [26] including the annotation file which contained only one gene model for each gene. The downloaded expression data were already normalized by Bioconductor (http://www.bioconductor.org/) R software. The final sample size for analysis was 18,445 genes, excluding two (2) genes from the coding sequence that only had 1 bp which was classified as an intron. The accession string and ID reference from the arrays were used to link the data together to create a master database of length and gene expression data for analysis.

The* Drosophila melanogaster* sequence data were downloaded from FlyBase website: http://www.flybase.org/. The raw CEL gene expression data files were downloaded from the NCBI GEO Datasets database (http://www.ncbi.nlm.nih.gov/geo/query/acc.cgi?acc=GSE42255) under series GSE42255 [27]. Affymetrix microarrays were used to analyse the adult* Drosophila* and the raw CEL files were normalized using the Bioconductor (http://www.bioconductor.org/) affy package in the R software environment. The annotation file was included and the Entrez UniGene name (GC numbers) and the ID from the platform data table were used to link the data together to create a master database of length and gene expression. The final sample size of unique genes was 3,290 for analysis.

The downloaded text files for each organism were cleaned using Visual Basic scripts and imported into MS Excel; all length data for both coding and noncoding sequences exclude introns. For each organism the gene expression experiments included multiple replicates of the control as well as abiotic stress conditions. For this study we have only reported on the control condition expression from the GEO Datasets for both organisms, to simplify the analysis reporting. Abiotic stress conditions will be investigated at a later stage.

### 2.2. Statistical Analysis

Pearson's correlation was used to test the gene expression data to determine the reliability of the control replicates. The *R* ^2^ value was found never below 0.95, demonstrating the accuracy and reproducibility of the raw data. Therefore, the mean of the results of the control biological replicas was used in the statistical analysis reporting. The gene expression measurements are represented by gene expression signal intensity.

In this study we are interested in whether the length of the coding and noncoding sequences has a significant impact on the probability distribution of the gene expression under control conditions. Quantiles are statistics that describe the subdivisions of a ranked set of data values into equal proportions. Divisions can be made in four parts corresponding to 25%, 50%, and 75% of the data. Firstly, to examine how the data behaves between the sequence length of each region and gene expression, the length data for each region (5′ UTR, CDS, and 3′ UTR) were split into 4 quartiles (groups 1, 2, 3, and 4).

**Figure 1:**
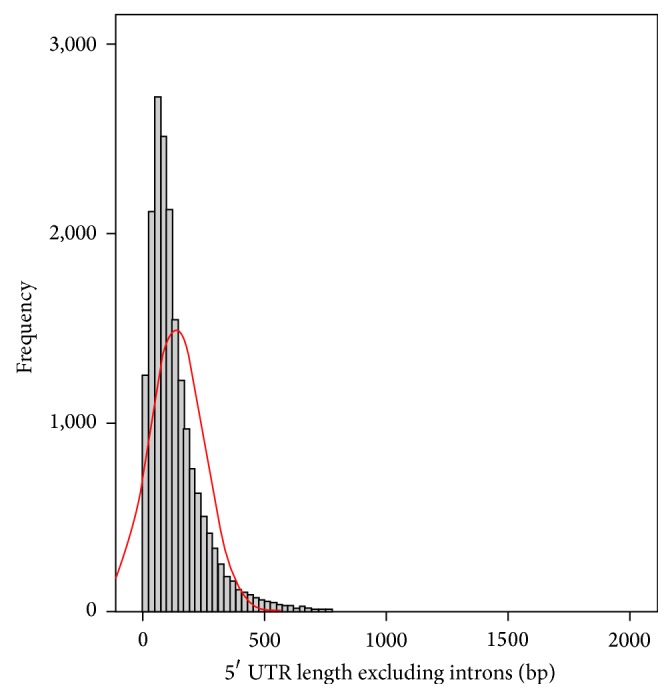
18,445 genes in* Arabidopsis thaliana* for the 5′ untranslated region (UTR) length, excluding introns. The distribution of this data is positively skewed (skewness = 2.511).

Strong skewness was identified in all the length datasets for each gene region. For example, the distribution of the 5′ UTR length without introns in* Arabidopsis thaliana* was positively skewed (skewness = 2.511) (Figure 1). Consequently, the Kruskal-Wallis nonparametric analysis method using SPSS version 19 (SPSS IBM, New York, USA) was applied to the data to determine whether there are differences between the quartile groups, in relation to gene expression and the length of the coding and noncoding regions. This test makes no assumptions about the distribution of the data:

(1) $$\begin{array}{c} K=\left(\;N-1\;\right)\frac{\sum_{i=1}^{g}n_{i}{\left({\bar{r}}_{i\cdot }-\bar{r}\right)}^{2}}{\sum_{i=1}^{g}\sum_{j=1}^{n_{i}}{\left(r_{ij}-\bar{r}\right)}^{2}}, \end{array}$$

where *n*_*i*_ is the number of observations in group *i* and *r*_*ij*_ is the rank (among all observations) of observation *j* from group *i*. *N* is the total number of observations across all groups. ${\bar{r}}_{i\cdot }=(\sum_{j=1}^{n_{i}}r_{ij})/n_{i}$ and $\bar{r}=\left(1/2\right)(N+1)$ is the average of all *r*_*ij*_.

### 2.3. Quantile Regression Analysis

The purpose of regression analysis is to expose the relationship between the independent variable (*x*) and dependent variables (*y*). Conditional quantile regression is useful in modelling the quantile value of the dependent variable on the independent variable. In this study, the dependent variable is represented by the log of gene expression, under control conditions, and the independent variable is represented by the log of the sequence length. The lengths considered include the coding and noncoding sequences (5′ UTR, CDS, and 3′ UTR). The model considered was linear and is represented by

(2) $$\begin{array}{c} \log Control=\beta_{0}+\beta_{1}\log x+\varepsilon . \end{array}$$

The quantile subsets used ranged from 0.1 to 0.9 in 0.1 increments. The log of the data was used to expand the data points for an enhanced view of the quantile regions. Regression analysis was performed in R.

## 3. Results

### 3.1. Length Subset Analysis

**Figure 2:**
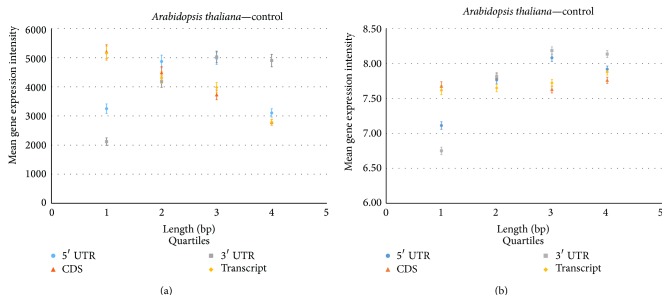
Relationship of gene expression in* Arabidopsis thaliana* within the coding and noncoding sequence regions. The gene expression intensity from GEO Datasets, GSE31488 (a) and GDS3933 (b), is plotted versus the quartile score for coding sequence, transcript, 5′ UTR and 3′ UTR regions. Each data point represents the mean for the samples in each quartile. Error bars represent standard error.

**Figure 3:**
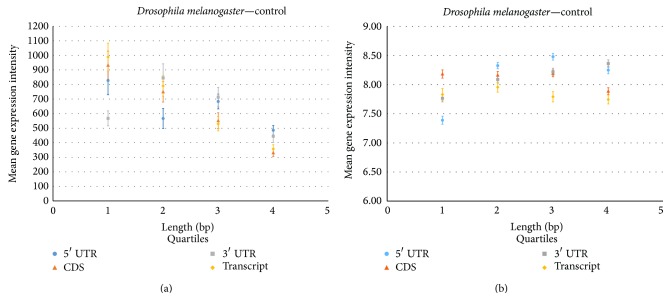
Relationship of gene expression in* Drosophila melanogaster* within coding and noncoding regions. The gene expression intensity from GEO Datasets, GSE42255 (a) and GSE36507 (b), is plotted versus the quartile score for coding sequence, transcript, 5′ UTR and 3′ UTR regions. Each data point represents the mean for the samples in each quartile. Error bars represent standard error.

To understand the relationship of the length of the coding and noncoding sequences and gene expression, the data of the lengths for each type of coding and noncoding regions were grouped into four quartile subsets, respectively. For each quartile subset (1, 2, 3, and 4), the gene expression data in each of these quartiles were averaged. Through the nonparametric analysis method, the mean of the gene expression conditional on the four quartile groups for each length region, respectively, was significantly different (*p* value < 0.000) (Figures 2(a) and 3(a)).

For* Arabidopsis* the coding sequences show a linear negative relationship between the four quartiles (groups 1–4) and their average gene expression intensity, indicating that as the length increases, the gene expression intensity decreases. This pattern is also seen in* Drosophila* (Figure 3(a)). The same pattern is also seen in the full transcript length, which follows the same negative relationship, in both the animal and plant species.

However, the noncoding sequences show dissimilar trends to the coding sequence. The relationship between the length of the 5′ UTR and gene expression intensity for* Arabidopsis* indicates a quadratic form, with an increase in length until the average gene expression intensity peaks for those genes in the 3rd group determined by the 3rd quartile and then starts to decrease (Figure 2(a)).

The pattern seen in the 3′ UTR length data was more positively correlated in relation to the average gene expression intensity, in contrast to the CDS and 5′ UTR sequence length. This pattern implies that as the length of the 3′ UTR increases (from 1 to 3318 base pairs) the gene expression intensity increases (Figure 2(a)).

Furthermore, in* Drosophila*, the noncoding sequences in relation to the average gene expression intensity varied considerably from* Arabidopsis*. The patterns showed a reversal in the 3′ and 5′ UTR sequence length in relation to the average gene expression. The 3′ UTR gene expression intensity increased until the 2nd quartile and then decreased at the 4th quartile, again showing signs of a nonlinear relationship. The pattern in the 5′ UTR for* Drosophila* was very distinctive, displaying a cubic polynomial pattern with one turning point (Figure 3(a)).

In summary, the findings based on the 4 quartile subsets show some variability between the coding and noncoding sequences as well as between animal and plant species. The quartile analysis indicates that the coding sequence is negatively correlated to the average gene expression intensity for both the animal and plant species. The full transcript sequence, which includes the flanking 5′ and 3′ UTRs, also shows negative correlation to the average gene expression intensity again in both species. However, when the gene is divided into coding and noncoding regions, differing patterns emerge from each of these gene regions in the plant and animal species. It is important to note that these gene region lengths do not include introns, the gene expression values are measured under control conditions, and the gene length and gene expression data for this analysis have not been transformed.

To determine the validity of the findings in the first set of gene expression experiments, a second set of gene expression data was downloaded from the GEO Datasets website. The raw CEL gene expression files were downloaded GDS3933 [28],* Arabidopsis*, and GSE36507,* Drosophila*, and normalised by MAS5 using R. The label and hybridization protocols for* Arabidopsis* varied between each experimental sample, the first sample using Agilent Low RNA Input Linear Amplification Kit and the second sample using GeneChip 3′ IVT Express Kit. In both samples, total RNA was extracted.

For* Drosophila* both gene expression samples used 7–9-day-old adults, with total RNA extraction. The labels used were biotin; however the protocols for labelling varied between the gene expression samples. Hybridization protocols followed similar methods. Length data and the master databases containing the length and gene expression data were generated with the same method as outlined in Methods above.

The quartile results show similar results to the first set of gene expression analyses. The noncoding sequences (5′ and 3′ UTRs) in both the animal and plant species displayed an increase in the first two quartiles and then decreased. However, for the coding sequence, there was not such a dramatic decline in gene expression from each quartile (Figures 2(b) and 3(b)).

To test the distribution of gene expression across the four quartile groups, nonparametric analysis was applied to the new gene expression samples. As seen in the previous example, the mean of the gene expression conditional on the four quartile groups for each length region, respectively, was significantly different (*p* value < 0.0000 at significance level 0.05) (Figures 2(b) and 3(b)).

For the experimental analyses with the quartile length subsets, it is difficult to achieve a general opinion on patterns observed in the coding and noncoding sequences in relation to gene expression. The data in the four subsets do not have sufficient resolution to determine accurately identifiable patterns in both the animal and plant species. However, based on the nonparametric analysis, both samples' results were unanimous in showing significant differences between the gene expression and the four quartile length groups. The results reported in the length subset analysis of this paper and the results on the relationship between gene expression intensity, and length in general, published in the literature, have directed us to employ a different analytical method to examine more precisely this relationship.

### 3.2. Quantile Regression Analysis

**Figure 4:**
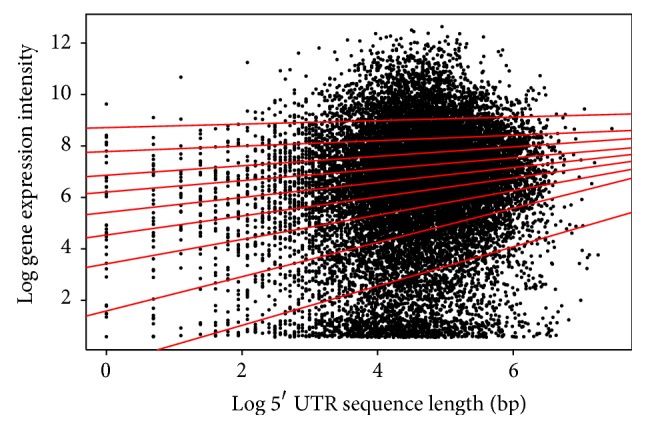
Quantile regression plot for* Arabidopsis thaliana* with quantiles range from 0.1 to 0.9 in increments of 0.1, respectively.

The log function was used to transform the data for an improved view of the quantile regions, a method not applied in the analysis above. Distinct patterns in the quantile regression for both the animal and plant species are evident in the analysis. Firstly, the length of the 5′ UTR and the gene expression in both* Arabidopsis* (Table 1/Figure 4) and* Drosophila* (Table 4/Figure 7) show a positive correlation in the majority of quantiles, indicating that as the length of the 5′ UTR increases gene expression increases. However, in the* Drosophila* at the 9th quantile, the pattern changes and shows a negative correlation, indicating that, in this quantile for the* Drosophila*, the 5′ UTR length increases as the gene expression decreases.

**Figure 5:**
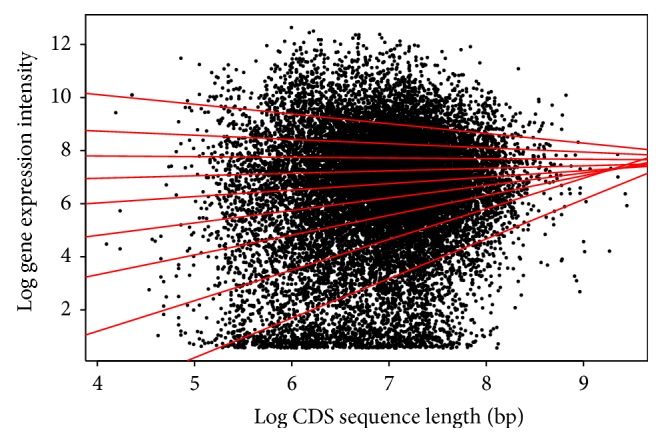
Quantile regression plot for* Arabidopsis thaliana* with quantiles range from 0.1 to 0.9 in increments of 0.1, respectively.

For the CDS length, each species shows a different pattern among the quantiles. For* Arabidopsis* (Table 2/Figure 5), the pattern shows a positive correlation for the first six (6) quantiles, and then from the 7th quantile there appears to be negative correlation. This would indicate that within the first six quantiles as the CDS length increases, the gene expression increases, and this is reversed past the 7th quantile. The* Drosophila* result (Table 5/Figure 8) in all quantiles shows negative correlation, indicating that as the CDS length increases, gene expression decreases. This shows two very distinctive patterns between the animal and plant species when the CDS is examined.

**Figure 6:**
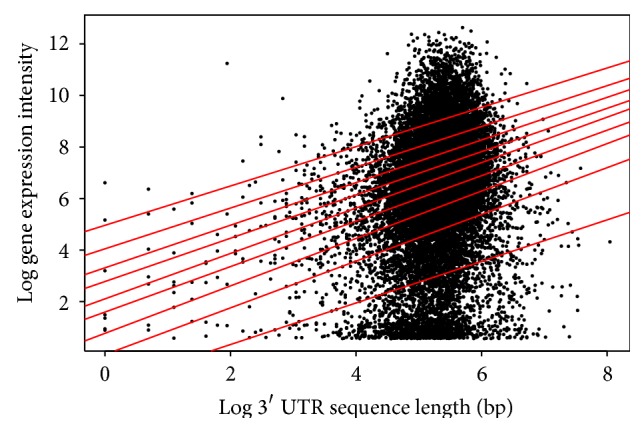
Quantile regression plot for* Arabidopsis thaliana* with quantiles range from 0.1 to 0.9 in increments of 0.1, respectively.

**Figure 7:**
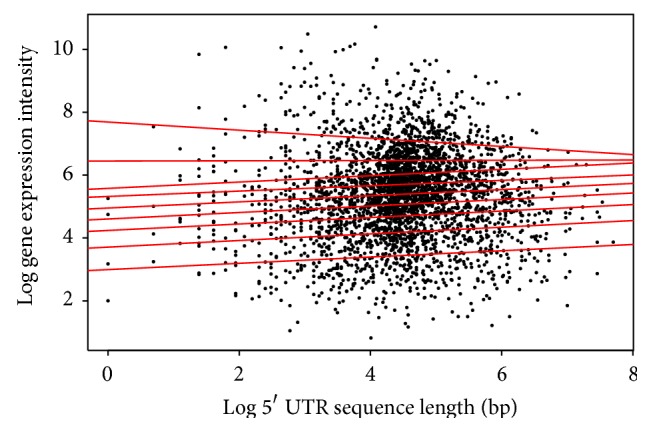
Quantile regression plot for* Drosophila melanogaster* with quantiles range from 0.1 to 0.9 in increments of 0.1, respectively.

**Figure 8:**
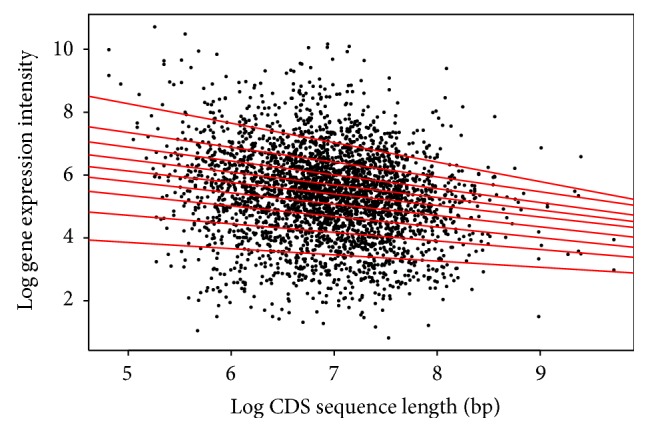
Quantile regression plot for* Drosophila melanogaster* with quantiles range from 0.1 to 0.9 in increments of 0.1, respectively.

**Figure 9:**
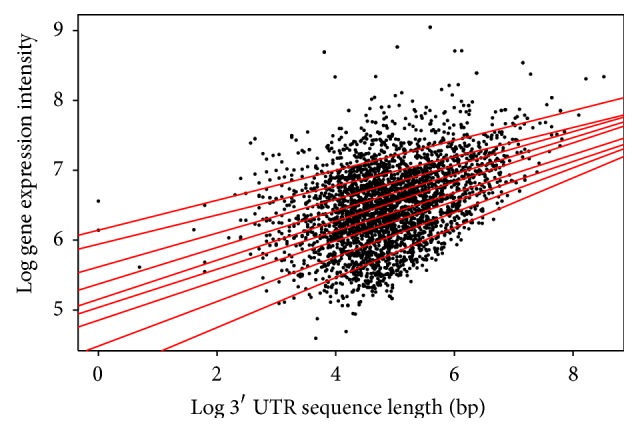
Quantile regression plot for* Drosophila melanogaster* with quantiles range from 0.1 to 0.9 in increments of 0.1, respectively.

Finally, for the 3′ UTR length, the interesting result for both* Arabidopsis* (Table 3/Figure 6) and* Drosophila* (Table 6/Figure 9) was that all quantiles showed positive correlation between the 3′ UTR length and gene expression. This suggests that as the 3′ UTR length increases, gene expression increases.

Overall, the CDS length and gene expression appeared dissimilar between the animal and plant species, with different patterns observed. However, when comparing the 5′ UTR and 3′ UTR lengths (noncoding regions of the gene) with gene expression data, similarities emerged.

**Figure 10:**
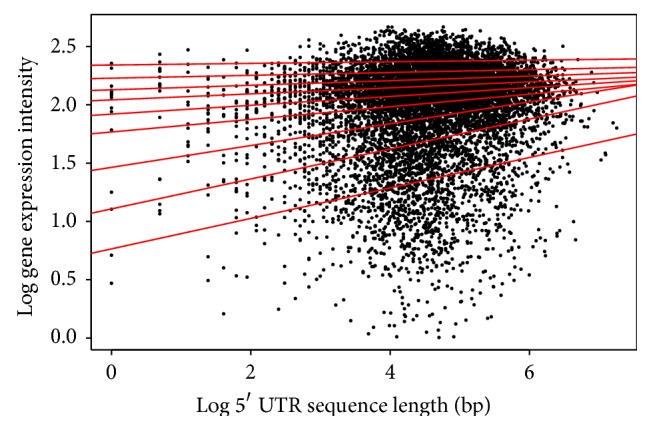
Quantile regression plot for* Arabidopsis thaliana* with quantiles range from 0.1 to 0.9 in increments of 0.1, respectively.

**Figure 11:**
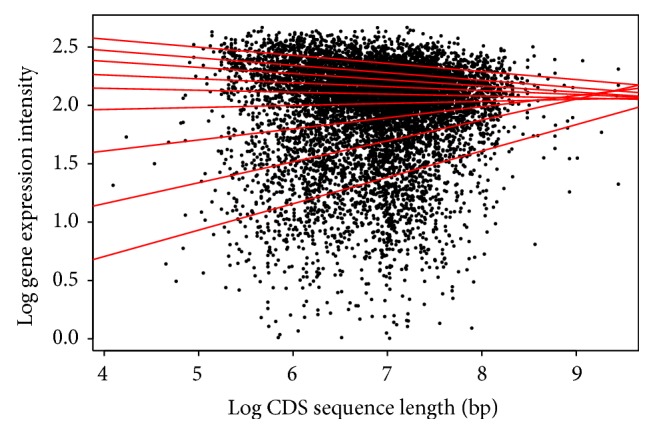
Quantile regression plot for* Arabidopsis thaliana* with quantiles range from 0.1 to 0.9 in increments of 0.1, respectively.

**Figure 12:**
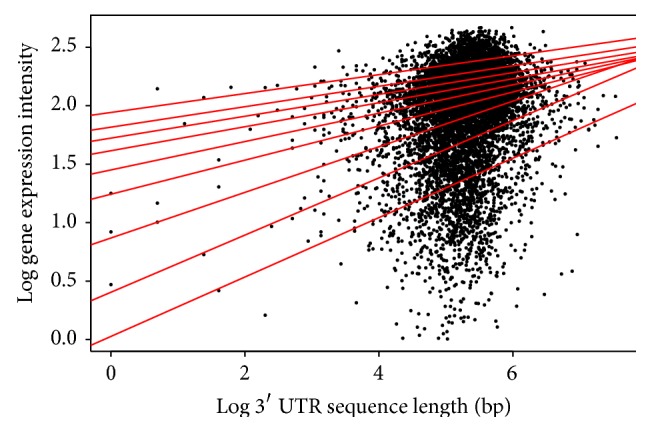
Quantile regression plot for* Arabidopsis thaliana* with quantiles range from 0.1 to 0.9 in increments of 0.1, respectively.

**Figure 13:**
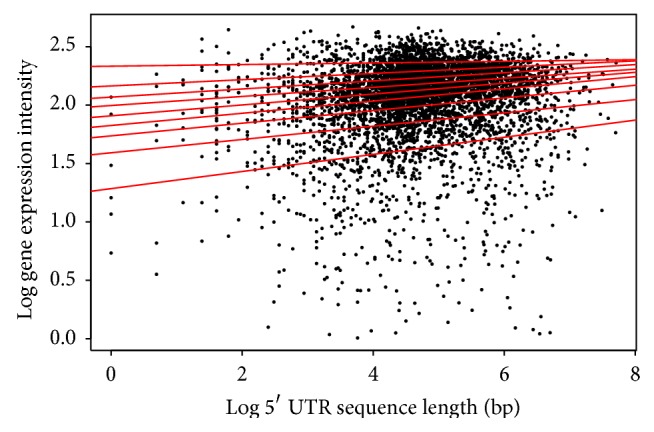
Quantile regression plot for* Drosophila melanogaster* with quantiles range from 0.1 to 0.9 in increments of 0.1, respectively.

**Figure 14:**
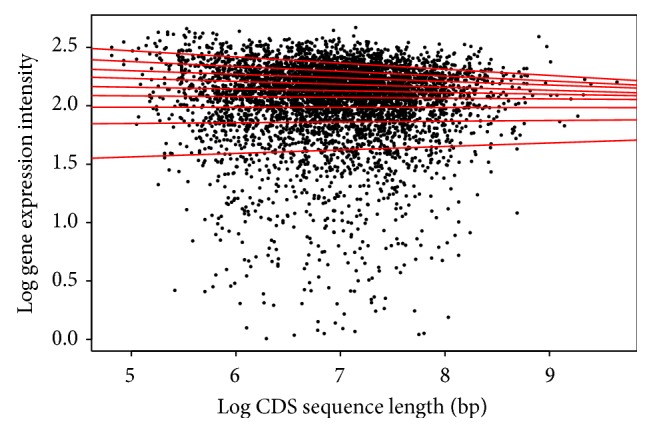
Quantile regression plot for* Drosophila melanogaster* with quantiles range from 0.1 to 0.9 in increments of 0.1, respectively.

**Figure 15:**
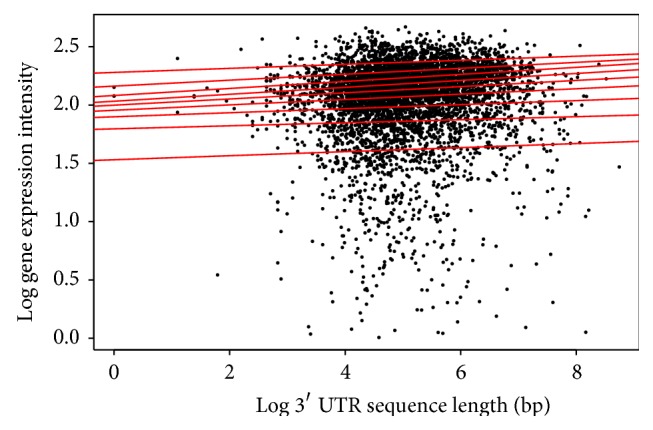
Quantile regression plot for* Drosophila melanogaster* with quantiles range from 0.1 to 0.9 in increments of 0.1, respectively.

The quantile regression statistical analyses were again applied to the second set of gene expression data to substantiate this method under different gene expression experiments. The results show very similar patterns to the previous gene expression experiment, indicating the model is robust in studying the relationship between gene expression and the length of coding and noncoding regions in different species (Tables 7–12/Figures 10–15). Both gene expression datasets showed statistical significance across all quantile groups, indicating a relationship between the coding and noncoding length and gene expression in animal and plant species.

The observed expression trends in both experimental datasets suggest that there are differences between animal and plant species when considering CDS length and that the noncoding regions show similar patterns of positive correlation to gene expression.

## 4. Discussion

We aimed to develop an understanding of the relationship between the coding and noncoding sequence length in association with gene expression between animal and plant species. In brief the findings from the quantile regression analysis suggest that (i) the patterns seen between the CDS length and gene expression intensity in* Arabidopsis* and* Drosophila* are different, the plant species showing both positive and negative correlations dependent on the quantile whilst the animal species showing a consistent negative correlation among all quantiles; (ii) in both the animal and plant species the 3′ UTR length and gene expression exhibit positive correlation.

The current research has confirmed our previous findings with the* Arabidopsis* [17] and is also consistent with previous research, where it was found that highly expressed genes have larger primary transcripts [15]. Extensive studies with* Arabidopsis* have inferred that multistimuli response genes (genes that are differentially expressed in response to a large number of different external stimuli) have significantly longer upstream intergenic regions and are generally shorter [29]. A more recent study investigating the translational efficiency in* Arabidopsis* has proposed that the sequence context immediately upstream from the AUG initiation codon in plant genes is critical in determining translational efficiency [30]. Other studies investigating the role of the 5′ UTR in translational regulation found that nucleotide composition, length, potential secondary structure, and the presence of uAUGs have a considerable effect on ribosome loading in* Arabidopsis* [31]. Furthermore, additional studies have focused on the GC content showing large variability among species, ~20 to 60% variation in eukaryotes [32]. Based on the findings from Duret and Stoletzki, GC3-rich genes tend to be shorter than GC3-poor genes [33, 34]. To investigate the hypothesis of synonymous codon usage (SCU), which is described as highly expressed genes undergoing stronger translational selection, for example, higher GC content, in seeded plants, Serres-Giardi et al. tested GC3-rich and GC-poor genes against expression. It was found that, in 154 plant species tested, expression was significantly and positively correlated with GC3 [35]. The results from these studies are interesting with respect to our results and may support and extend the understanding of gene architecture and gene expression in plants.

In addition, the patterns found in the coding sequences for* Drosophila* are consistent with previous research with animals. A study on* Gallus gallus* (chicken) found that the coding sequence length is negatively correlated with expression level [13] as shown in the* Drosophila* in this study. In other animal investigations, the research also reported that in highly expressed genes the length of the coding sequence and protein lengths were small [14, 36]. A popular bioinformatics technique used to detect subtle variations in sequences was used to identify differences between the 3′ UTR and protein coding sequences in the* Drosophila*. Interestingly, the study found greater number of segments in the 3′ UTR, suggesting greater functional complexity in the 3′ UTRs than in the coding sequence [37]. This could explain the differences in the CDS and 3′ UTR patterns found in this study. Genome size is also another important aspect in determining variability between organisms. A* Drosophila melanogaster* study has shown that genomes are subjected to constant change not only in their size but also in their composition [38].

Identification of similarities and differences in genomes, particularly between animals and plants that might result in speciation, has had a great deal of interest, with gene families, gene loss, and gene amplification being the focus of these studies [39]. The genomes of* Arabidopsis* and* Drosophila* are of similar size; however the number of genes identified varies, ~26,000 for* Arabidopsis* and ~14,000 for* Drosophila*. Differences start to emerge when gene families are examined;* Arabidopsis* appear to have 11,000 gene families, which have more than five members, in contrast to* Drosophila* which encode fewer genes [40]. Understanding the genome structure of these organisms before examining the finer details of the genome itself is an important strategy.

When the coding sequence is examined in association with gene expression there seems to be divergence in* Arabidopsis* and* Drosophila*, although we cannot yet conclude and refer in general to the difference between animal and plant genomes. Differences seen in the animal and plants species may be described by differences in life strategies [11]. Plant genomes appear much more dynamic [10], due to the sessile nature and response to adverse conditions through biochemical complexity and developmental plasticity [41]. In contrast, animal genomes are more conserved and stable, attributable to the ability to avoid adverse conditions [10]. There has been overwhelming evidence that natural selection appears to support the compactness of highly expressed genes in both animal and plant species [13, 16, 42–44]. These results may elucidate to the theory on reduction costs of energy with shorter proteins and sequences, contributing to minimizing the cost of synthesis [45]. However it is important to highlight that the length of the coding region is only one of several factors that contribute to the complex nature of natural selection, species complexity, and gene regulation.

Furthermore, the noncoding untranslated sequences have been identified as important components in the regulation of transcription and translation, influencing translation initiation, stability, elongation, and the termination of the mRNA translation [46]. Modification to the lengths of the 5′ UTR and 3′ UTR sequences may contribute to the selective constraints between animal and plants species and may be influenced by environmental conditions [47]. For the 3′ UTR regions, the results of this study have shown similarities in the patterns between* Arabidopsis* and* Drosophila*, that is, positive correlation between length and gene expression. This is in agreement with our previous research for* Arabidopsis* [17].

The regulation of many genes has been known to be controlled primarily by 3′ UTRs, particularly those involved in development [48]. Other research has found that there was positive correlation with transposon and simple sequence repeats (SSRs), with these elements affecting the length and variation of both the 5′ and 3′ UTRs [49]. Differing lengths of the untranslated regions could also be affected by either selection or genetic drift [47]. These results may enforce the concept that these untranslated regions are prone to a higher level of environmental and evolutionary constraints compared to the coding sequences and it is plausible that selection shapes these lengths. However, Chen et al. looked at over 15 species and found that the elongation of 5′ UTR alone cannot lead to the emergence of organismal complexity [47], indicating that the untranslated regions may not be a true indication of organism evolution, thus supporting the similarities found in this research in the untranslated regions.

Furthermore, recent experimental studies have shed light on the complex ceRNA network dynamics in prostate cancer using the alternative cleavage and polyadenylation (APA). This study concluded that long 3′ UTRs tend to harbour more microRNA response elements (MREs) which in turn would influence biological process when the 3′ UTR length is modified. The understanding of 3′ UTR shortening has great potential in creating prognostic markers for oncogene expression [50]. Other research in mammalian brain development proposes that lengthening of 3′ UTRs offers considerable versatility in biological processes [51]. The findings in this study have amplified the importance of the noncoding 5′ and 3′ UTR regions and have shown differences in these regions compared to the coding sequence.

At a global scale, the picture emerging is that animal and plant species show similarities and divergences when comparisons are made with gene expression and the length distributions of the coding and noncoding regions. However, studying the association between expression levels and length can be intricate to interpret, including sample size variation between organisms, statistical methodology, and data transformation. It was our intention to take advantage of available genomic data to identify general responses and relations. Using the available technologies and data our results have shown some interesting correlation between gene expression and the basic gene architecture, length, especially in the 3′ UTR region. Further research is required to explore more details in the gene length distribution variations of different genes and different organisms, including known highly expressed genes such as heat shock protein genes (HSPs).

## Figures and Tables

**Table 1:** Quantile regression analysis results on *Arabidopsis thaliana* between the log of 5′ UTR sequence length and the log of gene expression (GSE31488 gene expression experiment data).

Quantile		Value	Std. error	*t* value	Pr (>|*t*|)
0.1	Intercept	−0.49412	0.26154	−1.88925	0.05887
	Log 5′ UTR	0.76284	0.05660	13.47864	0.00000

0.2	Intercept	1.59094	0.19615	8.11066	0.00000
	Log 5′ UTR	0.66533	0.04014	16.57554	0.00000

0.3	Intercept	3.42035	0.14372	23.79799	0.00000
	Log 5′ UTR	0.47395	0.02935	16.14634	0.00000

0.4	Intercept	4.54025	0.11701	38.80151	0.00000
	Log 5′ UTR	0.36948	0.02413	15.30925	0.00000

0.5	Intercept	5.41919	0.10368	52.26962	0.00000
	Log 5′ UTR	0.29044	0.02129	13.64508	0.00000

0.6	Intercept	6.21008	0.09453	65.69373	0.00000
	Log 5′ UTR	0.22153	0.01970	11.24759	0.00000

0.7	Intercept	6.86249	0.09495	72.27477	0.00000
	Log 5′ UTR	0.18379	0.01959	9.38290	0.00000

0.8	Intercept	7.78214	0.09417	82.63627	0.00000
	Log 5′ UTR	0.10587	0.02002	5.28773	0.00000

0.9	Intercept	8.71224	0.13789	63.18310	0.00000
	Log 5′ UTR	0.06857	0.02834	2.41939	0.01556

**Table 2:** Quantile regression analysis results on *Arabidopsis thaliana* between the log of CDS sequence length and the log of gene expression (GSE31488 gene expression experiment data).

Quantile		Value	Std. error	*t* value	Pr (>|*t*|)
0.1	Intercept	−7.25569	0.44465	−16.31763	0.00000
	Log CDS	1.49091	0.06397	23.30774	0.00000

0.2	Intercept	−3.42118	0.35382	−9.66938	0.00000
	Log CDS	1.15369	0.04837	23.85127	0.00000

0.3	Intercept	0.34012	0.25491	1.33425	0.18214
	Log CDS	0.74619	0.03464	21.54367	0.00000

0.4	Intercept	2.92526	0.21057	13.89182	0.00000
	Log CDS	0.47024	0.02846	16.52017	0.00000

0.5	Intercept	5.05024	0.20765	24.32114	0.00000
	Log CDS	0.24374	0.02887	8.44371	0.00000

0.6	Intercept	6.58158	0.19429	33.87460	0.00000
	Log CDS	0.09393	0.02720	3.45279	0.00056

0.7	Intercept	7.89733	0.19186	41.16143	0.00000
	Log CDS	−0.02494	0.02698	−0.92442	0.35528

0.8	Intercept	9.37143	0.20560	45.58077	0.00000
	Log CDS	−0.15842	0.02889	−5.48414	0.00000

0.9	Intercept	11.57666	0.23543	49.17176	0.00000
	Log CDS	−0.36614	0.03348	−10.93737	0.01556

**Table 3:** Quantile regression analysis results on *Arabidopsis thaliana* between the log of 3′ UTR sequence length and the log of gene expression (GSE31488 gene expression experiment data).

Quantile		Value	Std. error	*t* value	Pr (>|*t*|)
0.1	Intercept	−1.28003	0.43043	−2.97380	0.00295
	Log 3′ UTR	0.80649	0.08240	9.78727	0.00000

0.2	Intercept	−0.05783	0.36157	−0.15994	0.87293
	Log 3′ UTR	0.90699	0.06794	13.34944	0.00000

0.3	Intercept	0.76806	0.22584	3.40086	0.00067
	Log 3′ UTR	0.92050	0.04268	21.56976	0.00000

0.4	Intercept	1.59305	0.20227	7.87587	0.00000
	Log 3′ UTR	0.88246	0.03802	23.21245	0.00000

0.5	Intercept	2.10509	0.16906	12.45189	0.00000
	Log 3′ UTR	0.88162	0.03187	27.66366	0.00000

0.6	Intercept	2.77838	0.16847	16.49139	0.00000
	Log 3′ UTR	0.84038	0.03151	26.66661	0.00000

0.7	Intercept	3.31070	0.15947	20.76044	0.00000
	Log 3′ UTR	0.82708	0.03010	27.48028	0.00000

0.8	Intercept	4.04752	0.19399	20.86466	0.00000
	Log 3′ UTR	0.79168	0.03630	21.81226	0.00000

0.9	Intercept	4.96045	0.15761	31.47265	0.00000
	Log 3′ UTR	0.76333	0.03044	25.07947	0.00000

**Table 4:** Quantile regression analysis results on *Drosophila melanogaster* between the log of 5′ UTR sequence length and the log of gene expression (GSE42255 gene expression experiment data).

Quantile		Value	Std. error	*t* value	Pr (>|*t*|)
0.1	Intercept	2.99298	0.24447	12.24295	0.00000
	Log 5′ UTR	0.09999	0.05405	1.85007	0.06439

0.2	Intercept	3.70770	0.18407	20.14305	0.00000
	Log 5′ UTR	0.10567	0.04101	2.57692	0.01001

0.3	Intercept	4.23852	0.16040	26.42456	0.00000
	Log 5′ UTR	0.10295	0.03436	2.99667	0.00275

0.4	Intercept	4.60339	0.13736	33.51435	0.00000
	Log 5′ UTR	0.10315	0.03002	3.43561	0.00060

0.5	Intercept	4.95174	0.12961	38.20391	0.00000
	Log 5′ UTR	0.09782	0.02782	3.51598	0.00044

0.6	Intercept	5.31990	0.11417	46.59480	0.00000
	Log 5′ UTR	0.08538	0.02580	3.30864	0.00095

0.7	Intercept	5.57721	0.14373	38.80300	0.00000
	Log 5′ UTR	0.10068	0.02962	3.39947	0.00068

0.8	Intercept	6.44681	0.17437	36.97142	0.00000
	Log 5′ UTR	0.00413	0.03571	0.11577	0.90784

0.9	Intercept	7.68730	0.22150	34.70589	0.00000
	Log 5′ UTR	−0.12948	0.04432	−2.92142	0.00351

**Table 5:** Quantile regression analysis results on *Drosophila melanogaster* between the log of CDS sequence length and the log of gene expression (GSE42255 gene expression experiment data).

Quantile		Value	Std. error	*t* value	Pr (>|*t*|)
0.1	Intercept	4.84616	0.57613	8.41160	0.00000
	Log CDS	−0.19783	0.08014	−2.46860	0.01361

0.2	Intercept	6.07890	0.44560	13.64200	0.00000
	Log CDS	−0.27198	0.06264	−4.34194	0.00001

0.3	Intercept	7.03180	0.34350	20.47108	0.00000
	Log CDS	−0.33594	0.04954	−6.78088	0.00000

0.4	Intercept	7.59350	0.32054	23.68971	0.00000
	Log CDS	−0.36004	0.04521	−7.96411	0.00000

0.5	Intercept	7.95531	0.28030	28.38178	0.00000
	Log CDS	−0.36548	0.04029	−9.07219	0.00000

0.6	Intercept	8.49326	0.26724	31.78114	0.00000
	Log CDS	−0.40083	0.03742	−10.71079	0.00000

0.7	Intercept	9.08676	0.28572	31.80286	0.00000
	Log CDS	−0.44001	0.04007	−10.98103	0.00000

0.8	Intercept	9.70859	0.34560	28.09197	0.00000
	Log CDS	−0.47106	0.04905	−9.60348	0.00000

0.9	Intercept	11.36497	0.42722	26.60187	0.00000
	Log CDS	−0.61925	0.05964	−10.38366	0.00000

**Table 6:** Quantile regression analysis results on *Drosophila melanogaster* between the log of 3′ UTR sequence length and the log of gene expression (GSE42255 gene expression experiment data).

Quantile		Value	Std. error	*t* value	Pr (>|*t*|)
0.1	Intercept	4.03542	0.08225	49.06280	0.00000
	Log 3′ UTR	0.35700	0.01546	23.09641	0.00000

0.2	Intercept	4.48795	0.08257	54.35470	0.00000
	Log 3′ UTR	0.31840	0.01614	19.72846	0.00000

0.3	Intercept	4.85297	0.06886	70.47384	0.00000
	Log 3′ UTR	0.28407	0.01273	22.30680	0.00000

0.4	Intercept	5.03650	0.06313	79.77872	0.00000
	Log 3′ UTR	0.27350	0.01290	21.20361	0.00000

0.5	Intercept	5.15329	0.05986	86.09041	0.00000
	Log 3′ UTR	0.27983	0.01173	23.85901	0.00000

0.6	Intercept	5.37147	0.06384	84.13507	0.00000
	Log 3′ UTR	0.26200	0.01199	21.84826	0.00000

0.7	Intercept	5.61639	0.06897	81.43580	0.00000
	Log 3′ UTR	0.24204	0.01329	18.21441	0.00000

0.8	Intercept	5.94198	0.07576	78.43611	0.00000
	Log 3′ UTR	0.20878	0.01507	13.85648	0.00000

0.9	Intercept	6.14204	0.09774	62.84273	0.00000
	Log 3′ UTR	0.21414	0.01879	11.39432	0.00000

**Table 7:** Quantile regression analysis results on *Arabidopsis thaliana* between the log of 5′ UTR sequence length and the log of gene expression (GDS3933 gene expression experiment data).

Quantile		Value	Std. error	*t* value	Pr (>|*t*|)
0.1	Intercept	0.76451	0.06266	12.20094	0.00000
	Log 5′ UTR	0.13061	0.01399	9.33390	0.00000

0.2	Intercept	1.10719	0.05709	19.39225	0.00000
	Log 5′ UTR	0.12840	0.01203	10.67434	0.00000

0.3	Intercept	1.46345	0.04631	31.60327	0.00000
	Log 5′ UTR	0.09359	0.00930	10.06655	0.00000

0.4	Intercept	1.76655	0.02690	65.66343	0.00000
	Log 5′ UTR	0.05411	0.00541	10.00009	0.00000

0.5	Intercept	1.91977	0.02053	93.49082	0.00000
	Log 5′ UTR	0.03789	0.00419	9.03235	0.00000

0.6	Intercept	2.04182	0.01720	118.70693	0.00000
	Log 5′ UTR	0.02580	0.00356	7.23725	0.00000

0.7	Intercept	2.12815	0.01635	130.17843	0.00000
	Log 5′ UTR	0.01962	0.00340	5.77582	0.00000

0.8	Intercept	2.22796	0.01691	131.74460	0.00000
	Log 5′ UTR	0.01224	0.00355	3.44724	0.00057

0.9	Intercept	2.34085	0.01530	153.04522	0.00000
	Log 5′ UTR	0.00692	0.00338	2.04747	0.04064

**Table 8:** Quantile regression analysis results on *Arabidopsis thaliana* between the log of CDS sequence length and the log of gene expression (GDS3933 gene expression experiment data).

Quantile		Value	Std. error	*t* value	Pr (>|*t*|)
0.1	Intercept	−0.19868	0.15081	−1.31744	0.18772
	Log CDS	0.22601	0.02155	10.48761	0.00000

0.2	Intercept	0.44103	0.11273	3.91235	0.00009
	Log CDS	0.17926	0.01574	11.39117	0.00000

0.3	Intercept	1.22926	0.08138	15.10571	0.00000
	Log CDS	0.09495	0.01100	8.63142	0.00000

0.4	Intercept	1.89539	0.04992	37.97150	0.00000
	Log CDS	0.01738	0.00666	2.61013	0.00907

0.5	Intercept	2.21277	0.03694	59.90485	0.00000
	Log CDS	−0.01660	0.00509	−3.25824	0.00113

0.6	Intercept	2.39485	0.03619	66.17765	0.00000
	Log CDS	−0.03350	0.00508	−6.59286	0.00000

0.7	Intercept	2.58925	0.03022	85.68926	0.00000
	Log CDS	−0.05279	0.00423	−12.48248	0.00000

0.8	Intercept	2.72644	0.03013	90.48563	0.00000
	Log CDS	−0.06385	0.00433	−14.75102	0.00000

0.9	Intercept	2.84588	0.03396	83.79063	0.00000
	Log CDS	−0.06942	0.00490	−14.18200	0.00000

**Table 9:** Quantile regression analysis results on *Arabidopsis thaliana* between the log of 3′ UTR sequence length and the log of gene expression (GDS3933 gene expression experiment data).

Quantile		Value	Std. error	*t* value	Pr (>|*t*|)
0.1	Intercept	0.026500	0.131040	0.202270	0.839710
	Log 3′ UTR	0.254060	0.024960	10.177990	0.000000

0.2	Intercept	0.404410	0.120200	3.364370	0.000770
	Log 3′ UTR	0.244440	0.022430	10.895530	0.000000

0.3	Intercept	0.868450	0.077930	11.144220	0.000000
	Log 3′ UTR	0.195080	0.014290	13.648320	0.000000

0.4	Intercept	1.242830	0.048620	25.563150	0.000000
	Log 3′ UTR	0.145700	0.008920	16.330550	0.000000

0.5	Intercept	1.450620	0.040690	35.652010	0.000000
	Log 3′ UTR	0.121350	0.007440	16.313380	0.000000

0.6	Intercept	1.614860	0.033970	47.538100	0.000000
	Log 3′ UTR	0.102630	0.006330	16.220570	0.000000

0.7	Intercept	1.722880	0.028790	59.848790	0.000000
	Log 3′ UTR	0.093360	0.005410	17.256770	0.000000

0.8	Intercept	1.816540	0.034440	52.742530	0.000000
	Log 3′ UTR	0.087470	0.006430	13.599130	0.000000

0.9	Intercept	1.942250	0.034110	56.939020	0.000000
	Log 3′ UTR	0.080820	0.006430	12.572240	0.000000

**Table 10:** Quantile regression analysis results on *Drosophila melanogaster* between the log of 5′ UTR sequence length and the log of gene expression (GSE36507 gene expression experiment data).

Quantile		Value	Std. error	*t* value	Pr (>|*t*|)
0.1	Intercept	1.28497	0.06779	18.95641	0.00000
	Log 5′ UTR	0.07333	0.01374	5.33611	0.00000

0.2	Intercept	1.59194	0.04478	35.54779	0.00000
	Log 5′ UTR	0.05687	0.00909	6.25711	0.00000

0.3	Intercept	1.73679	0.02932	59.23962	0.00000
	Log 5′ UTR	0.05415	0.00614	8.82520	0.00000

0.4	Intercept	1.82558	0.02286	79.85883	0.00000
	Log 5′ UTR	0.05229	0.00465	11.24806	0.00000

0.5	Intercept	1.90830	0.01715	111.28562	0.00000
	Log 5′ UTR	0.04659	0.00343	13.60097	0.00000

0.6	Intercept	1.99646	0.01926	103.68233	0.00000
	Log 5′ UTR	0.03888	0.00388	10.01241	0.00000

0.7	Intercept	2.06843	0.01618	127.85772	0.00000
	Log 5′ UTR	0.03482	0.00320	10.86927	0.00000

0.8	Intercept	2.16414	0.01809	119.63192	0.00000
	Log 5′ UTR	0.02680	0.00357	7.50320	0.00000

0.9	Intercept	2.33342	0.02376	98.21304	0.00000
	Log 5′ UTR	0.00693	0.00468	1.48068	0.13875

**Table 11:** Quantile regression analysis results on *Drosophila melanogaster* between the log of CDS sequence length and the log of gene expression (GSE36507 gene expression experiment data).

Quantile		Value	Std. error	*t* value	Pr (>|*t*|)
0.1	Intercept	1.41356	0.14825	9.53503	0.00000
	Log CDS	0.02975	0.02106	1.41256	0.15784

0.2	Intercept	1.81659	0.08947	20.30396	0.00000
	Log CDS	0.00639	0.01276	0.50060	0.61667

0.3	Intercept	1.99122	0.06902	28.85156	0.00000
	Log CDS	−0.00075	0.00975	−0.07669	0.93887

0.4	Intercept	2.11710	0.04834	43.79536	0.00000
	Log CDS	−0.00646	0.00688	−0.93966	0.34744

0.5	Intercept	2.23380	0.03819	58.48915	0.00000
	Log CDS	−0.01500	0.00536	−2.79907	0.00514

0.6	Intercept	2.36145	0.03839	61.51257	0.00000
	Log CDS	−0.02540	0.00533	−4.76824	0.00000

0.7	Intercept	2.45361	0.03457	70.97384	0.00000
	Log CDS	−0.03070	0.00489	−6.27982	0.00000

0.8	Intercept	2.58566	0.03616	71.50751	0.00000
	Log CDS	−0.04147	0.00506	−8.19982	0.00000

0.9	Intercept	2.73350	0.03934	69.48483	0.00000
	Log CDS	−0.05262	0.00554	−9.49641	0.00000

**Table 12:** Quantile regression analysis results on *Drosophila melanogaster* between the log of 3′ UTR sequence length and the log of gene expression.

Quantile		Value	Std. error	*t* value	Pr (>|*t*|)
0.1	Intercept	1.53054	0.08075	18.95458	0.00000
	Log 3′ UTR	0.01735	0.01533	1.13164	0.25784

0.2	Intercept	1.79567	0.04229	42.46001	0.00000
	Log 3′ UTR	0.01303	0.00840	1.55044	0.12110

0.3	Intercept	1.89908	0.03645	52.09484	0.00000
	Log 3′ UTR	0.01735	0.00720	2.40887	0.01604

0.4	Intercept	1.95487	0.02397	81.56678	0.00000
	Log 3′ UTR	0.02306	0.00468	4.92510	0.00000

0.5	Intercept	1.99859	0.01908	104.74509	0.00000
	Log 3′ UTR	0.02653	0.00382	6.95355	0.00000

0.6	Intercept	2.03241	0.01928	105.41200	0.00000
	Log 3′ UTR	0.02976	0.00376	7.90712	0.00000

0.7	Intercept	2.08155	0.01642	126.79634	0.00000
	Log 3′ UTR	0.03018	0.00309	9.75937	0.00000

0.8	Intercept	2.16193	0.01891	114.31813	0.00000
	Log 3′ UTR	0.02546	0.00356	7.15683	0.00000

0.9	Intercept	2.27943	0.02397	95.09426	0.00000
	Log 3′ UTR	0.01735	0.00458	3.79243	0.00015
